# Sex differences in symptomatology in people with schizophrenia and other psychotic disorders: protocol for a systematic review and pairwise meta-analysis of observational studies

**DOI:** 10.1192/bjo.2022.596

**Published:** 2022-11-08

**Authors:** Marta Ferrer-Quintero, Marina Verdaguer-Rodriguez, Marina Esteban Sanjusto, Clara Serra-Arumí, Judith Usall, Susana Ochoa, Irene Bighelli, Helena García-Mieres

**Affiliations:** Institut de Recerca Sant Joan de Déu, Barcelona, Spain; Centro de Investigación Biomédica en Red de Salud Mental (CIBERSAM), Madrid, Spain; and Departament de Psicología Social i Quantitativa, Universitat de Barcelona, Spain; Institut de Recerca Sant Joan de Déu, Barcelona, Spain; Institut de Recerca Sant Joan de Déu, Barcelona, Spain; and Centro de Investigación Biomédica en Red de Salud Mental (CIBERSAM), Madrid, Spain; Department of Psychiatry and Psychotherapy, School of Medicine, Technical University of Munich, Germany; Institut de Recerca Sant Joan de Déu, Barcelona, Spain; Centro de Investigación Biomédica en Red de Epidemiología y Salud Pública (CIBERESP), Madrid, Spain; Health Services Research Unit, Institut Hospital del Mar de Investigacions Médiques (IMIM), Barcelona, Spain; and Department of Medicine and Health Sciences, Universitat Pompeu Fabra, Barcelona, Spain

**Keywords:** Sex differences, schizophrenia, first-episode psychosis, ultra-high risk for psychosis, symptoms

## Abstract

Sex differences in symptomatology in people with psychosis have been studied extensively in recent decades. Although studies have pointed to such differences, to date there is no review that has performed a systematic search and quantitative synthesis. In this paper, we describe the protocol for a pairwise meta-analysis comparing a range of symptom outcome measures between men and women diagnosed with a psychotic spectrum disorder at different stages of the disorder (PROSPERO registration number CRD42021264942). In August 2021 we conducted systematic searches of PsychInfo, PubMed, Web of Science, Scopus and Dialnet to identify observational studies that report data on symptoms for males and females separately. Two independent reviewers will conduct literature searches, select studies, extract data, assess the risk of bias and assess outcome quality. To assess the effect size of all outcome measures, we will conduct pairwise meta-analysis using random-effects models. The quality of studies will be evaluated using a National Heart, Lung and Blood Institute's quality assessment tool and the confidence in the results will be evaluated using the GRADE tool. Meta-regression and sensitivity analyses will be conducted to assess the robustness of the findings. No ethical problems are foreseen. Results from this study will be published in peer-reviewed journals and presented at relevant conferences.

Schizophrenia and other psychotic disorders present considerable heterogeneity in several of their core features. Since the first conceptualisations of these, researchers have noticed that their clinical presentation and course seem to be different in males and females. Evidence suggests that females have a later age at onset and better prognosis,^1,2^ but whether males and females with psychosis present differences in symptoms is not yet clear.

Several narrative reviews published in recent years have examined the characterisation of sex differences in a broad array of outcomes in people with psychosis and have linked these differences to the contribution of sex hormones and molecular mechanisms.^3^ The meta-analysis described in the protocol presented here will focus on symptoms. The symptoms present in people with psychosis are usually categorised into a range of five to eight domains: positive symptoms (e.g. delusions and hallucinations), negative symptoms (e.g. anhedonia), disorganised thought and behaviour, cognitive symptoms and affective outcomes such as depression and mania. Although narrative reviews suggest that women tend to express more depressive symptoms and men experience more negative symptoms,^4,5^ the results of different studies are still heterogeneous and do not permit the drawing of sound conclusions. In people considered at ultra-high risk for psychosis, a review concluded that men had more severe negative symptoms before the onset of illness. However, the methodological limitations of the studies again did not allow the establishment of clear conclusions.^6^ In contrast, a recent review argues that the differences could be explained by comorbidities and illness behaviours such as substance misuse, which is more frequent in men and not controlled for in all studies.^1^

Therefore, evidence is still inconclusive and limited by methodological shortcomings. Although there are systematic reviews and meta-analyses examining the role of sex in aspects of psychosis such as age at onset of the disorder, duration of untreated psychosis and cognitive functioning,^2,7,8^ none of them focused on sex differences in symptoms.

As a result of these inconsistencies, it is still unclear in which domain(s) of clinical symptoms men and women have a different expression, nor in which they do not have true differences. However, given that sex differences in psychosis have been described in all aspects of the disease, understanding how the disorder is expressed in males and females could not only clarify aetiological aspects of psychosis, but also identify therapeutic targets and inform the development of accurate and directed interventions. Furthermore, these achievements will contribute to reducing the still present sex bias in mental health research.^9^

To overcome this gap in the current knowledge, we will perform a pairwise meta-analysis comparing data on a broad array of symptoms relevant to the expression of psychosis in men and women with a psychosis spectrum disorder at different stages of the illness.

Personalised treatment approaches in other fields of psychiatry are proving to be successful, and tailoring the treatment symptom targets of psychosis for each person, considering the sex of the individual, will be a crucial step forward.^1^

## Aim and objectives

Our aim is to statistically synthesise and estimate whether males and females at different stages of schizophrenia and other psychotic disorders (ultra-high risk mental state, first-episode psychosis and established illness) present differences in symptoms. These will include:

- psychotic symptomatology, including positive, negative and disorganised symptoms;
- depressive symptomatology and other symptoms relevant to the person's functioning, including mania and anxiety symptoms; symptoms relevant to psychosis that can be identified will also be collected and analysed;
- control for relevant variations in study methodology and for variables that might influence the clinical expression of psychosis.

## Method

### Design

We have developed the methods for this systematic review and meta-analysis following the guidelines of the Preferred Reporting Items for Systematic Review and Meta-Analysis Protocols (PRISMA-P) checklist.^10^ This systematic review and meta-analysis is registered in the PROSPERO database (registration number: CRD42021264942). The PROSPERO record will be updated should any changes be made to the protocol.

Ethics approval and patient consent are not required as all data were sourced from previously reported studies.

### Eligibility criteria

#### Participants

This meta-analysis will include male and female individuals aged 16–65 years with a diagnosis of schizophrenia or other psychotic disorder (schizophrenia, schizophreniform, schizoaffective disorder, delusional disorder, brief psychotic disorder, recent-onset psychosis, ultra-high risk for psychosis) irrespective of the diagnostic criteria used, following the strategy of the Cochrane Schizophrenia Group. The Cochrane Schizophrenia Group suggests that specific diagnostic criteria – such as those of ICD-11 or DSM-5 – are not accurately used in routine clinical practice. Therefore, including studies that do not use these systems should increase generalisability and representativeness.^11^ Moreover, our purpose is to examine the duality male/female as determined by sex chromosomes, but we cannot control the use of ‘gender’ and ‘sex’ in the studies we are including in the review.

Studies with participants with non-psychotic disorders will be included only if individuals with a diagnosis of a psychotic disorder represent more than 80% of the sample.

We will exclude studies in which all participants, by study inclusion criteria, (a) are acutely ill, (b) have a comorbid pathology or psychosis secondary to another psychiatric or medical diagnosis (e.g. dual pathology, bipolar disorder with psychotic symptoms, psychotic depression, obsessive–compulsive disorder comorbid with psychosis), (c) have a concomitant medical illness or (d) have dementia or intellectual disability (premorbid IQ < 70).

For the purposes of the review we define recent-onset psychosis as a first diagnosis of schizophrenia or other psychotic disorder within the preceding 5 years, according to research done in our group^12^ and established operational definitions.^13^

#### Studies

We will include both cross-sectional and longitudinal observational studies. To be included, we require that a study presents data about outcomes separately for males and females. For the case of longitudinal studies or clinical trials, we will only include their baseline data.

To reduce the risk of ‘language bias’,^14^ the included languages will be English, Spanish, Italian, German and Chinese. We will consider studies irrespective of setting (in-patients and out-patients), nationality and ethnicity. Grey literature will be included where identified.

#### Outcomes

As primary outcomes, we will consider data on positive, negative, depressive and disorganised symptomatology and general psychopathology measured using published and validated scales such as the Positive and Negative Syndrome Scale (PANSS), Brief Psychiatric Rating Scale (BPRS), Scale for the Assessment of Positive Symptoms (SAPS), Scale for the Assessment of Negative Symptoms (SANS), Beck Depression Inventory (BDI) and Calgary Depression Inventory (CDI).

As secondary outcomes, we will consider data on other symptoms of interest in the study of psychosis (e.g. mania, anxiety). As with primary outcomes, we will include secondary outcomes measured using rating scales published in peer-reviewed journals, such as the Beck Anxiety Inventory (BAI) and the Young Mania Rating Scale (YMRS).

### Search strategy and information sources

#### Electronic searches

The PsychInfo, PubMed, Web of Science, Scopus and Dialnet databases were searched in August 2021 without restrictions for publication period. The final search strategy for PubMed appears in the Appendix. The full search strategies for the different databases are shown the supplementary material available at https://dx.doi.org/10.1192/bjo.2022.596. The date of the last search update will be provided in the final publication.

#### Reference lists and other sources

We will inspect (a) previous narrative reviews concerning sex differences in psychotic disorders in a broad array of outcomes and (b) the reference lists of our included studies, to check for additional studies that could meet our inclusion criteria not found by the electronic search.

#### Identification and selection of studies

Studies identified using electronic and manual searches will be exported and listed in the Picoportal software;^15^ duplicates will be excluded. Determining eligibility for the inclusion will comprise the following two stages.

1. Screening: two authors (M.F.-Q. and M.E.S.) will independently check titles and abstracts identified in the literature searches. Records that do not meet inclusion criteria will be excluded. Disagreement will be resolved by discussion. If there is remaining doubt, we will obtain the full article for further inspection.
2. Eligibility: in the inspection of full articles, the same two authors (M.F.-Q. and M.E.S.) will independently assess them for eligibility. Disagreements will be resolved with a third author (H.G.-M.) or, when this is not possible, by contacting the study authors.

### Data extraction

Data will be independently extracted by two authors working independently using a standard data collection form. Results will be compared and inconsistencies resolved by discussing with a third author acting as an arbitrator or, when this is not possible, by contacting the study authors. Collected data will include:

- study citation, year of study, year of publication, setting and country
- sample characteristics (diagnosis, diagnostic system, number of males, number of females, age, ethnicity, stage of disorder, sample source, location); when available, we will collect other sociodemographic and descriptive variables of interest, such as age at onset, premorbid adjustment and working status
- study design
- outcome measures.

We will contact study authors to ask for data missing in the reports.

### Study quality assessment

We will use the National Heart, Lung and Blood Institute (NHLBI) quality assessment tool for observational cohort and cross-sectional studies.^16^ Users of this tool must focus on key attributes relevant to the internal validity of the studies, rather than on numerical scores. The following questions illustrate some of the domains considered by the tool:

- Was the research question or objective in this paper clearly stated?
- Was the study population clearly specified and defined?
- Was the participation rate of eligible persons at least 50%?
- Were all the participants selected or recruited from the same or similar populations (including the same time period)? Were inclusion and exclusion criteria for being in the study prespecified and applied uniformly for all participants?
- Was a sample size justification, power description or variance and effect estimation provided?
- Were the outcome measures (dependent variables) clearly defined, valid, reliable and implemented consistently across all study participants?
- Were the outcome assessors not involved in the care of the patient?
- Were key potential confounding variables measured and adjusted statistically for their effect on the relationship between exposure(s) and outcome(s)?

A judgement on the study quality is made based on the following three categories: ‘Good methodological quality’, ‘Fair methodological quality’ and ‘Poor methodological quality’. Two review authors will independently assess the quality of the selected studies and any disagreement will be resolved through discussion. If necessary, the senior author (H.G.-M.) will act as an arbitrator. Our group has extensive experience using this tool for assessing risk of bias in previous systematic reviews.^17^ Effects of studies with a given rating of ‘Poor’ in the overall domain will be analysed by excluding them in sensitivity analyses.

### Assessing overall quality of evidence: adaptation of GRADE

We will use an adaptation of the Grading of Recommendations Assessment, Development and Evaluation (GRADE) approach^18^ to evaluate the quality of evidence at the outcome level for observational studies following procedures used by other authors.^19^ Observational studies are usually rated down for quality using GRADE. However, as all our included studies will be observational, we will initially rate all outcomes as high quality and then downgrade them based on five main criteria: risk of bias, imprecision, indirectness, heterogeneity and publication bias. Two authors will evaluate the confidence in the outcome independently, and a senior author will act as an arbitrator. The authors involved will complete the GRADE online learning modules before starting the evaluation (https://cebgrade.mcmaster.ca/).

### Data analysis

#### Characteristics of the included studies

We will generate descriptive statistics and study sample characteristics across all eligible studies, describing clinical and methodological variables such as sample distribution (number of males and females), age, diagnoses, duration of the disorder, country, setting and scales used to measure symptoms.

#### Pairwise meta-analysis: data synthesis

As we expect most studies to report means and standard deviations, we will calculate standardised mean differences (s.m.d.) with 95% confidence intervals for each outcome.^20^ If an asset-based outcome has negative valence, we will recode the means (multiplied by −1) so that the valences coincide. For studies with more than one scale in the same outcome group, we will convert mean values for each of these measures to a single mean value for the intervention and control groups respectively. We will compute the variance of the mean between scales within the same outcome grouping using Borenstein et al's method.^21^ If means or standard deviations are not available to calculate effect size and associated standard error we will estimate it/them from other reported statistics (e.g. *t*, *f*) or contact study authors for this information. If we find studies in which symptomatology is treated as a binary variable, odds ratios (OR) will be calculated as an index of effect size.

We will perform series of pairwise meta-analyses by separating studies according to the stage of the disorder (ultra-high risk for psychosis, recent-onset psychosis and established psychosis) and separated by symptom domain. If there are not enough studies available for meta-analysis in a particular symptom domain for one of the stages of the disorder, these studies will be presented narratively in the form of systematic review. As heterogeneity is likely, we will use a random-effects model.

#### Assessment of heterogeneity

Statistical heterogeneity will be assessed using Cochran's *Q*, *I^2^* and *τ^2^*. Cochran's *Q* is a chi-squared distributed measure of weighted squared deviations. It can be converted into a *P*-value and is the usual heterogeneity test statistic. The principal advantage of the *I^2^* statistic, the proportion of the observed variance reflecting real differences in effect size, is that it can be calculated and compared across meta-analyses of different sizes, of different types of study and using different types of outcome data.^22^ Finally, *τ^2^* is the random-effects variance of the true effect sizes.

#### Investigation of heterogeneity: meta-regression and sensitivity analysis

As we expect some degree of heterogeneity in our outcomes, we will explore the following potential effect modifiers of our symptomatology outcomes by meta-regression (for continuous variables) and subgroup analyses (for dichotomous variables) if there are ten or more studies that include these factors:

- year of publication of the study
- country of the study: high-income versus low- and middle-income countries
- setting: in-patients versus out-patients (on enrolment in the study)
- sample size
- diagnostic system, for example manualised diagnostic criteria (such as DSM or ICD) versus clinical diagnosis (where no diagnostic manual is referred to)
- relevant sociodemographic characteristics that might affect symptom presentation. Given that sex differences in outcomes in people with psychosis vary depending on the individual's age,^23^ one of the main characteristics that will be controlled for will be the age of the participants in the studies. Other characteristics to be controlled for may include age at onset, duration of illness, duration of untreated psychosis, premorbid adjustment, substance misuse, marital status, work status and dose of antipsychotic medication.

We will perform two types of sensitivity analysis if there are enough studies:

- exclusion of studies characterised as being of poor methodological quality (as assessed using the NHLBI assessment tool)
- exclusion of studies where the median or mean age of the participants is over 45 years.

#### Publication bias

To assess small study effects and publication bias, we will use contour-enhanced funnel plots and Begg & Mazumdar^24^ tests by outcome valence if ten or more studies are included.

#### Statistical software

All analyses will be done using the R package ‘meta’.^25,26^ The procedures described here follow steps similar to those used in previous meta-analyses done by the corresponding author (H.G.-M.).^27^

## Discussion

We have described the study protocol of a systematic review and pairwise meta-analysis comparing data on a broad array of symptoms relevant to the expression of psychosis in men and women. The aim of the study is to statistically synthesise and estimate whether males and females at different stages of schizophrenia and other psychotic disorders (ultra-high risk mental state, first-episode psychosis and established illness) present differences in symptoms.

### Strengths and limitations of the planned review

Our main strength is that this study would be the first systematic review and meta-analytic quantification of the role of sex in the expression of symptomatology in people with psychosis, overcoming the limitations of previous narrative reviews on the topic, thus providing the most reliable approach to evidence synthesis.

However, this review also has limitations. First, meta-analyses are not bias-free: reviewer selection bias and publication bias need to be considered. We will attempt to limit such biases by conducting an exhaustive systematic literature search, by including studies published in many languages with no limit on date and state of publication and by empirically examining publication bias. Second, as our meta-analysis will depend on data previously assessed in the included studies, it is possible that not all our outcome variables of interest will be examined. Third, we will combine data from multiple observational studies with differing inclusion criteria, target populations, countries, settings and symptom domains. However, the generalisability of the findings might still be limited to individuals who voluntarily participate in clinical studies.

### Dissemination

Findings will be published in peer-reviewed scientific journals and the data-set will be made publicly available on the Open Science Framework (identifier: DOI 10.17605/OSF.IO/82YS6, https://osf.io/82ys6/).

### Implications

The clinical expression of symptoms in people with psychosis has long been considered to be influenced by the sex of the individual. However, the robustness of this finding needs to be subjected to systematic review and the extent of the effect measured using meta-analytic procedures. Our proposed review will act as a definite investigation of this phenomenon. In light of this review, further development of the understanding of psychosis and tailoring of clinical practice using a sex-based and phase-specific approach may well be warranted.

## Data Availability

Data availability is not applicable to this article as no new data were created or analysed in this report.

## Supplementary material

For supplementary material accompanying this paper visit http://doi.org/10.1192/bjo.2022.596. click here to view supplementary material

## Appendix

### Final search strategy for PubMed

1. (‘schizophrenia spectrum and other psychotic disorders’[MeSH Terms] OR schizophren*[All Fields] OR ‘psychotic’[All Fields] OR ‘psychosis’[All Fields] OR ‘psychoses’ [All Fields]) OR ((‘high risk’[All Fields] OR ‘At-risk’[All Fields]) AND (‘Schizophrenia Spectrum and Other Psychotic Disorders’[MeSH Terms] OR (‘psychotic’[All Fields] AND ‘disorders’[All Fields]) OR ‘psychotic disorders’[All Fields] OR ‘psychoses’[All Fields] OR ‘psychotic’[All Fields] OR ‘psychotics’[All Fields] OR ‘schizophrenia’[All Fields] OR ‘schizophrenias’[All Fields] OR ‘schizophrenic’[All Fields] OR ‘schizophrenics’[All Fields] OR ‘Mental state’[All Fields]) OR ((prodrom*[All Fields]) AND (‘Schizophrenia Spectrum and Other Psychotic Disorders’[MeSH Terms] OR (‘psychotic’[All Fields] AND ‘disorders’[All Fields]) OR ‘psychotic disorders’[All Fields] OR ‘psychosis’[All Fields] OR ‘psychose’[All Fields] OR ‘psychoses’[All Fields] OR ‘schizophrenia’[All Fields] OR ‘schizophrenias’[All Fields])))
2. (‘sex characteristics’[MeSH Terms]) OR (‘sex’[All Fields] AND ‘characteristics’[All Fields]) OR (sex characteristics[All Fields]) OR (gender characteristics[All Fields]) OR (‘gender’[All Fields] AND ‘differences’[All Fields]) OR (gender differences[All Fields]) OR (‘sex’[All Fields] AND ‘differences’[All Fields]) OR (sex differences[All Fields]) OR (‘gender bias’[All Fields]) OR (‘sex bias’ [All Fields]) OR (gender bias[All Fields]) OR (sex bias[All Fields])
3. (‘Prodromal Symptoms’[MeSH Terms]) OR (‘Depressive symptoms’) OR (depress*) OR (‘Hallucinations’ [MeSH Terms]) OR (‘Delusions’ [MeSH Terms]) OR (delusions[All Fields]) OR (delusional [All Fields]) OR (hallucinations [All Fields]) OR (positive symptom[All Fields]) OR (negative symptom[All Fields]) OR (positive symptoms[All Fields]) OR (negative symptoms [All Fields]) OR (positive symptomatology[All Fields]) OR (negative symptomatology[All Fields]) OR (social withdrawal[All Fields]) OR (symptomatic[All Fields]) OR (emotional withdrawal[All Fields]) OR (blunted affect[All Fields]) OR (alogia[All Fields]) OR (avolition[All Fields]) OR (deficit syndrome[All Fields]) OR (‘disorganized’[All Fields]) OR (disorgani*[All Fields]) OR (‘clinical’[All Fields]) OR (‘course’[All Fields]) OR (‘symptoms’[All Fields]) OR (‘symptom’[All Fields]) OR (symptom dimension[All Fields]) OR (symptom dimensions[All Fields])
