# Supplementary material for: Sex differences in symptomatology in people with schizophrenia and other psychotic disorders: protocol for a systematic review and pairwise meta-analysis of observational studies
Source: BJPsych Open. 2022 Nov 8;8(6):e194. doi: 10.1192/bjo.2022.596 (PMC9707498; doi:10.1192/bjo.2022.596)
Supplement: Supplementary file 1 [file S2056472422005968sup001.pdf]

Supplementary material

Gender differences in symptomatology in people with schizophrenia and other psychotic disorders: protocol for a systematic review and pairwise meta-analysis of observational studies

Marta Ferrer-Quintero, Marina Verdaguer-Rodriguez, Clara Serra-Arumí, Marina Esteban Sanjusto, Susana Ochoa, Irene Bighelli, Judith Usall, Helena García-Mieres.

Corresponding author: Helena García-Mieres

Contents

Appendix 1: PRISMA-P checklist
Apprendix 2: Database search strategy

Appendix 1: PRISMA-P checklist

PRISMA-P (Preferred Reporting Items for Systematic review and Meta-Analysis Protocols) 2015 checklist: recommended items to address in a systematic review protocol*

Section and topic	Item No	Checklist item	Page
**ADMINISTRATIVE INFORMATION**			
Title:			
Identification	1a	Identify the report as a protocol of a systematic review	1
Update	1b	If the protocol is for an update of a previous systematic review, identify as such	NA
Registration	2	If registered, provide the name of the registry (such as PROSPERO) and registration number	3
Authors:			
Contact	3a	Provide name, institutional affiliation, e-mail address of all protocol authors; provide physical mailing address of corresponding author	1
Contributions	3b	Describe contributions of protocol authors and identify the guarantor of the review	14
Amendments	4	If the protocol represents an amendment of a previously completed or published protocol, identify as such and list changes; otherwise, state plan for documenting important protocol amendments	NA
Support:			
Sources	5a	Indicate sources of financial or other support for the review	14
Sponsor	5b	Provide name for the review funder and/or sponsor	14
Role of sponsor or funder	5c	Describe roles of funder(s), sponsor(s), and/or institution(s), if any, in developing the protocol	14
**INTRODUCTION**			
Rationale	6	Describe the rationale for the review in the context of what is already known	5
Objectives	7	Provide an explicit statement of the question(s) the review will address with reference to participants, interventions, comparators, and outcomes (PICO)	6
**METHODS**			
Eligibility criteria	8	Specify the study characteristics (such as PICO, study design, setting, time frame) and report characteristics (such as years considered, language, publication status) to be used as criteria for eligibility for the review	7
Information sources	9	Describe all intended information sources (such as electronic databases, contact with study authors, trial registers or other grey literature sources) with planned dates of coverage	8
Search strategy	10	Present draft of search strategy to be used for at least one electronic database, including planned limits, such that it could be repeated	Table 1
Study records:			
Data management	11a	Describe the mechanism(s) that will be used to manage records and data throughout the review	9
Selection process	11b	State the process that will be used for selecting studies (such as two independent reviewers) through each phase of the review (that is, screening, eligibility and inclusion in meta-analysis)	9

Data collection process	11c	Describe planned method of extracting data from reports (such as piloting forms, done independently, in duplicate), any processes for obtaining and confirming data from investigators	9
Data items	12	List and define all variables for which data will be sought (such as PICO items, funding sources), any pre-planned data assumptions and simplifications	9
Outcomes and prioritization	13	List and define all outcomes for which data will be sought, including prioritization of main and additional outcomes, with rationale	8
Risk of bias in individual studies	14	Describe anticipated methods for assessing risk of bias of individual studies, including whether this will be done at the outcome or study level, or both; state how this information will be used in data synthesis	10
Data synthesis	15a	Describe criteria under which study data will be quantitatively synthesised	11
	15b	If data are appropriate for quantitative synthesis, describe planned summary measures, methods of handling data and methods of combining data from studies, including any planned exploration of consistency (such as I^2, Kendall's τ)	11
	15c	Describe any proposed additional analyses (such as sensitivity or subgroup analyses, meta-regression)	12
	15d	If quantitative synthesis is not appropriate, describe the type of summary planned	NA
Meta-bias(es)	16	Specify any planned assessment of meta-bias(es) (such as publication bias across studies, selective reporting within studies)	13
Confidence in cumulative evidence	17	Describe how the strength of the body of evidence will be assessed (such as GRADE)	10

*** It is strongly recommended that this checklist be read in conjunction with the PRISMA-P Explanation and Elaboration (cite when available) for important clarification on the items. Amendments to a review protocol should be tracked and dated.** **The copyright for PRISMA-P (including checklist) is held by the PRISMA-P Group and is distributed under a Creative Commons Attribution Licence 4.0.**

From: Shamseer L, Moher D, Clarke M, Ghersi D, Liberati A, Petticrew M, Shekelle P, Stewart L, PRISMA-P Group. Preferred reporting items for systematic review and meta-analysis protocols (PRISMA-P) 2015: elaboration and explanation. BMJ. 2015 Jan 2;349(jan02 1):g7647.

2. Database search strategies

3.1. Search on 1st August 2021

We searched the following resources on 1st August 2021 with the inclusion and exclusion criteria explained in the full manuscript:

- PsychInfo
- PubMed
- Web of Science
- Scopus
- Dialnet.

3.2. Search strategy of Psychinfo

(ti(psychosis OR psychotic OR schizophrenia OR schizophr* OR “ultra high risk of psychosis” OR “first episode psychosis” OR “psychosis spectrum disorders” OR psychoses) OR ab(psychosis OR psychotic OR schizophrenia OR schizophr* OR “ultra high risk of psychosis” OR “first episode psychosis” OR “psychosis spectrum disorders” OR psychoses) OR MA(psychosis OR psychotic OR “psychosis spectrum disorders” OR schizophr*))
AND (ti(“gender differences” OR “sex differences” OR “gender” OR “sex”) OR ab(“gender differences” OR “sex differences” OR gender OR sex) OR tx(“gender differences” OR “sex differences” OR “gender” OR “sex”) OR MA(“human sex differences” OR “gender differences” OR “sex differences”)
AND (ti(symptoms OR "positive symptoms" OR "negative symptoms" OR sympt* OR "clinical course" OR "clinical" OR outcome* OR "hallucinations" OR "delusions" OR delusi* OR "disorganised" OR disorgan* OR "deficit syndrome") OR ab(symptoms OR "positive symptoms" OR "negative symptoms" OR sympt* OR "clinical course" OR "clinical" OR outcome* OR "hallucinations" OR "delusions" OR delusi* OR "disorganised" OR disorgan* OR "deficit syndrome" OR "avolition" OR "social withdrawa" l OR "blunted affect" OR symptomatology OR symptom* OR "depression" OR depress* OR "general symptoms" OR "general psychopathology" OR "clinical course" OR outcom* OR "symptom dimensions" OR "avolition" OR "deficit syndrome" OR "emotional withdrawal" OR symptom* OR "psychotic symptoms") OR (tx(symptoms OR "positive symptoms" OR "negative symptoms" OR sympt* OR "clinical course" OR "clinical" OR outcome* OR "hallucinations" OR "delusions" OR delusi* OR "disorganised" OR disorgan* OR "deficit syndrome" OR "avolition" OR "social withdrawa" l OR "blunted affect" OR symptomatology OR symptom* OR "depression" OR depress* OR "general symptoms" OR "general psychopathology" OR "clinical course" OR outcom* OR "symptom dimensions" OR "avolition" OR "deficit syndrome" OR "emotional withdrawal" OR symptom* OR "psychotic symptoms"))

3.2. Search strategy of PubMed

("schizophrenia spectrum and other psychotic disorders"[MeSH Terms] OR schizophren*[All Fields] OR "psychotic"[All Fields] OR "psychosis"[All Fields] OR “psychoses” [All Fields]) OR (("high risk"[All Fields] OR "At-risk"[All Fields]) AND (“Schizophrenia Spectrum and Other Psychotic Disorders"[MeSH Terms] OR ("psychotic"[All Fields] AND "disorders"[All Fields]) OR "psychotic disorders"[All Fields] OR "psychoses"[All Fields] OR "psychotic"[All Fields] OR "psychotics"[All Fields] OR "schizophrenia"[All Fields] OR "schizophrenias"[All Fields] OR "schizophrenic"[All Fields] OR "schizophrenics"[All Fields] OR "Mental state"[All Fields]) OR ((prodrom*[All Fields]) AND ("Schizophrenia Spectrum and Other Psychotic

Disorders"[MeSH Terms] OR ("psychotic"[All Fields] AND "disorders"[All Fields]) OR "psychotic disorders"[All Fields] OR "psychosis"[All Fields] OR "psychose"[All Fields] OR "psychoses"[All Fields] OR "schizophrenia"[All Fields] OR "schizophrenias"[All Fields])))
AND
("sex characteristics"[MeSH Terms]) OR ("sex"[All Fields] AND "characteristics"[All Fields]) OR (sex characteristics[All Fields]) OR (gender characteristics[All Fields])
OR ("gender"[All Fields] AND "differences"[All Fields]) OR (gender differences[All Fields]) OR ("sex"[All Fields] AND "differences"[All Fields]) OR (sex differences[All Fields]) OR (“gender bias”[All Fields]) OR (“sex bias” [All Fields]) OR (gender bias[All Fields]) OR (sex bias[All Fields])

AND
(“Prodromal Symptoms”[MeSH Terms]) OR (“Depressive symptoms”) OR (depress*) OR (“Hallucinations” [MeSH Terms]) OR (“Delusions” [MeSH Terms]) OR (delusions[All Fields]) OR (delusional [All Fields]) OR (hallucinations [All Fields]) OR (positive symptom[All Fields]) OR (negative symptom[All Fields]) OR (positive symptoms[All Fields]) OR (negative symptoms [All Fields]) OR (positive symptomatology[All Fields]) OR
(negative symptomatology[All Fields]) OR (social withdrawal[All Fields]) OR (symptomatic[All Fields]) OR (emotional withdrawal[All Fields]) OR (blunted affect[All Fields]) OR (alogia[All Fields]) OR (avolition[All Fields]) OR (deficit syndrome[All Fields]) OR (“disorganized”[All Fields]) OR (disorgani*[All Fields]) OR (“clinical”[All Fields]) OR (“course”[All Fields]) OR (“symptoms”[All Fields]) OR (“symptom”[All Fields]) OR (symptom dimension[All Fields]) OR (symptom dimensions[All Fields])

3.3. Search strategy of Web of Science

(TI=(psychosis OR “schizophrenia” OR "first episode psychosis" OR schizophr* OR “psychosis spectrum disorders” OR psychoses OR “ultra high risk of psychosis”) OR AB=(psychosis OR “schizophrenia” OR "first episode psychosis" OR schizophr* OR “psychosis spectrum disorders” OR psychoses OR “ultra high risk of psychosis”)) AND (TI= (sympt* OR “positive symptoms” OR “negative symptoms” OR depress*) OR AB=(“positive symptoms” OR “negative symptoms” OR “symptomatology” OR symptom* OR “depression” OR depress* OR "general symptoms" OR "general psychopathology" OR "clinical course" OR outcom* OR "symptom dimensions" OR “symptom dimensions” OR "deficit syndro me" OR "emotional withdrawal" OR “avolition” OR “social withdrawal”)) AND (TI=("gender differences" OR "sex differences" OR gender OR sex OR women OR male OR fem ale OR men) OR AB=(“gender differences” OR “sex differences” OR “sex characteristics” OR “g ender characteristics” OR “women” OR “men” OR “male” OR “female”))

3.4. Search strategy of Scopus

((TITLE ("ultra AND high AND risk AND of AND psychosis") OR TITLE ("early AND psychosis") OR TITLE ("first AND episode AND psychosis") OR TITLE ("psychosis AND spectrum AND disorders") OR TITLE (psychosis OR psychotic OR schizophr* OR prodom*)) AND TITLE ("gender differences" OR "sex differences" OR gender OR sex OR "sex characteristics" OR "gender characteristics" OR male OR female OR women OR men) AND (TITLE ("positive AND symptoms") OR TITLE ("negative AND symptoms") OR TITLE ("clinical AND course") OR TITLE ("deficit AND syndrome") OR TITLE ("experiential AND symptom") OR TITLE ("expressive AND symptom") OR TITLE (

"experiential AND deficit") OR TITLE ("expressive AND deficit") OR TITLE ("social AND withdrawal") OR TITLE ("blunted AND affect") OR TITLE ("general AND symptoms") OR TITLE ("general AND psychopathology") OR TITLE ("symptom AND dimensions") OR TITLE ("emotional AND withdrawal") OR TITLE ("psychotic AND symptoms") OR TITLE (
symptom* OR clinical OR outcome* OR hallucinations OR delusi* OR disorgan* OR a volition OR depression OR depress*))) OR ((ABS ("ultra AND high AND risk AND of AND psychosis") OR ABS ("early AND psychosis") OR ABS ("first AND episode AND psychosis") OR ABS ("psychosis AND spectrum AND disorders") OR ABS (schizophr* OR prodom*)) AND ABS ("gender differences" OR "sex differences" OR gender OR sex OR "sex characteristics" OR "gender characteristics") AND (ABS ("positive AND symptoms") OR ABS ("negative AND symptoms") OR ABS ("clinical AND course") OR ABS ("deficit AND syndrome") OR ABS ("experiential AND symptom") OR ABS ("expressive AND symptom") OR ABS ("experiential AND deficit") OR ABS ("expressive AND deficit") OR ABS ("social AND withdrawal") OR ABS ("blunted AND affect") OR ABS ("general AND symptoms") OR ABS ("general AND psychopathology") OR ABS ("symptom AND dimensions") OR ABS ("emotional AND withdrawal") OR ABS ("psychotic AND symptoms") OR ABS (
symptom* OR clinical OR outcome* OR hallucinations OR delusi* OR disorgan* OR a volition OR depression OR depress*))) OR ((AUTHKEY ("ultra AND high AND risk AND of AND psychosis") OR AUTHKEY ("early AND psychosis") OR AUTHKEY ("first AND episode AND psychosis") OR AUTHKEY ("psychosis AND spectrum AND disorders") OR AUTHKEY (psychosis OR psychotic OR schizophr* OR prodom*)) AND AUTHKEY ("gender differences" OR "sex differences" OR gender OR sex OR "sex characteristics" OR "gender characteristics" OR male OR female OR women OR men) AND (AUTHKEY ("positive AND symptoms") OR AUTHKEY ("negative AND symptoms") OR AUTHKEY ("clinical AND course") OR AUTHKEY ("deficit AND syndrome") OR AUTHKEY ("experiential AND symptom") OR AUTHKEY ("expressive AND symptom") OR AUTHKEY ("experiential AND deficit") OR AUTHKEY ("expressive AND deficit") OR AUTHKEY ("social AND withdrawal") OR AUTHKEY ("blunted AND affect") OR AUTHKEY ("general AND symptoms") OR AUTHKEY ("general AND psychopathology") OR AUTHKEY ("symptom AND dimensions") OR AUTHKEY ("emotional AND withdrawal") OR AUTHKEY ("psychotic AND symptoms") OR AUTHKEY (
symptom* OR clinical OR outcome* OR hallucinations OR delusi* OR disorgan* OR a volition OR depression OR depress*)))

3.5. Search strategy of Dialnet

(psicosis OR “psicótico*” OR “esquizofren*” OR “estado mental de algo riesgo” OR “prodom*” OR “esquizoafectivo” OR “trastornos del espectro psicótico” OR “primer episodio psicótico”)
AND
(“diferencias de género” OR “diferencia de género” OR “diferencias de sexo” OR género OR sexo OR “características de género”)
AND
(“síntomas positivos” OR “síntomas negativos” OR “síntoma*” OR “curso clínico” OR alucinaciones OR delirios OR delirante OR desorganizado OR “abulia” OR “anhedonia” OR

“retraimiento social” OR “afecto aplanado” OR depresi* OR “síntomas generales” OR psicopatología OR “psicopatología general” OR “dimesiones de síntomas” OR “síntomas psicóticos”)
